# MRI-Determined Tumor Contact Area as a Predictor of Pathological Extraprostatic Extension in Clinical T2 Prostate Cancer

**DOI:** 10.1155/proc/9165949

**Published:** 2025-10-26

**Authors:** Masashi Tsujimoto, Yuta Inoue, Hideto Taga, Yumiko Saito, Masatomo Kaneko, Masatsugu Miyashita, Takeshi Yamada, Yasuhiro Yamada, Takashi Ueda, Atsuko Fujihara, Takumi Shiraishi, Masayoshi Okumi, Fumiya Hongo, Eiichi Konishi, Kaori Yamada, Kei Yamada, Osamu Ukimura

**Affiliations:** ^1^Department of Urology, Kyoto Prefectural University of Medicine, Kyoto, Japan; ^2^Department of Surgical Pathology, Kyoto Prefectural University of Medicine, Kyoto, Japan; ^3^Department of Radiology, Kyoto Prefectural University of Medicine, Kyoto, Japan

## Abstract

**Objectives:**

To assess the validity of magnetic resonance imaging-determined tumor contact area (MRI-TCA) as a predictive factor for pathological extraprostatic extension (EPE) in cT2N0M0 prostate cancer patients.

**Methods:**

We retrospectively analyzed 72 cT2N0M0 prostate cancer patients who underwent multiparametric MRI (mpMRI) followed by robot-assisted laparoscopic prostatectomy (RARP) between February 2014 and April 2021. Patients whose MRI-based index lesion did not match the pathological specimens were excluded. MRI-TCA was approximated using an elliptical shape and calculated by two different methods: MRI-TCA1: Calculated using the tumor contact length (TCL) in the axial plane and the longer TCL in either the sagittal or coronal plane, capturing tumor dimensions across two planes. MRI-TCA2: Calculated using the TCL in the axial plane and tumor thickness derived from MRI slice data, reflecting the tumor's contact area within the MRI volume. We compared postoperative prostate-specific antigen (PSA) recurrence-free survival by stratifying patients based on the optimal thresholds of MRI-TCL, MRI-TCA1, MRI-TCA2, pathological-TCL, and pathological-TCA.

**Results:**

Sixteen patients (22.2%) were pathologically positive for EPE. MRI-TCL, MRI-TCA1, and MRI-TCA2 were significantly greater in patients with EPE-positive (EPE+) tumors than in those with EPE-negative (EPE−) tumors (*p* < 0.0001, *p* < 0.0001, and *p* = 0.0026, respectively). No statistically significant differences were found between MRI-TCL and MRI-TCA1 (*p* = 0.914) or between MRI-TCL and MRI-TCA2 (*p* = 0.112) in predicting pathological EPE. A significant difference in postoperative PSA recurrence rate was observed in the stratified analysis based on pathological-TCA (*p* = 0.022).

**Conclusion:**

Both MRI-TCA1 and MRI-TCA2 are clinically accessible and effective parameters for predicting pathological EPE in cT2N0M0 prostate cancer patients. However, neither method demonstrated clear superiority over MRI-TCL. Pathological-TCA was shown to be a significant predictor of both pathological EPE and postoperative PSA recurrence.

## 1. Introduction

Extraprostatic extension (EPE) in prostate cancer refers to the spread of cancer cells beyond the prostate gland margin. EPE is associated with positive surgical margins for cancer and is considered a significant risk factor for biological recurrence after radical prostatectomy (RP) in prostate cancer patients [1, 2]. While macroscopic EPE can typically be diagnosed by detecting abnormal signs in the prostate capsule on MRI, microscopic EPE is more challenging to identify due to the resolution limits of MRI and the small size (at least a micrometer) of cancer cells. We previously reported that ultrasonography- or MRI-determined tumor contact length (TCL) (defined as the length of cancer in contact with the prostate capsule) predicts the likelihood of microscopic EPE [3, 4]. Reliable assessment of microscopic EPE is particularly beneficial for cT2 patients diagnosed by mpMRI.

Given that the risk of EPE may correlate with the extent of cancer contact with the prostate margin, measuring the two-dimensional ‘area' of contact (tumor contact areas [TCA]) with the prostate capsule may provide a more accurate assessment than measuring the one-dimensional ‘length' (TCL). TCA, which reflects the cancer's contact surface area, has the potential to predict extraprostatic extension more accurately than TCL. Previous studies have suggested that the contact surface area of prostate cancer on mpMRI, calculated using 3D-conversion software, may be a superior predictor of EPE [5, 6].

**Figure 1:**
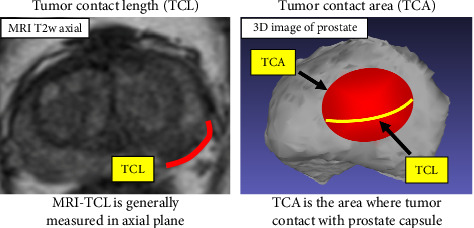
TCL and TCA. TCL is defined as the maximum length of the contour of index lesion which contact with the prostatic capsule. In our study, MRI-TCL was measured in axial plane. TCA is the area where tumor contact with prostate capsule.

In this study, we evaluated the validity of MRI-determined TCA (MRI-TCA), measured solely on the two-dimensional axial plane using simpler calculation methods (Figure 1). MRI-TCA does not require specialized image processing or 3D conversion software, making it a cost-effective and clinically applicable predictor of EPE+. This method can be implemented at any institution capable of measuring lengths on MRI images, offering a simple, versatile, and easily adoptable approach for predicting EPE.

## 2. Materials and Methods

Multiparametric MRI was performed using a 3 T magnetic field strength and a pelvic phased-array coil MR system (MAGNETOM Skyra, Siemens, Erlangen, Germany). T1-weighted, T2-weighted, diffusion-weighted, and dynamic gadolinium contrast-enhanced imaging sequences, including the calculation of apparent diffusion coefficient maps, were acquired.

We retrospectively collected data from prostate cancer patients who underwent RARP between February 2014 and April 2021 at our institution. All patients had at least one MRI-visible, biopsy-proven tumor (index tumor) that was diagnosed as organ-confined (cT2).

Inclusion Criteria: (i) Patients who underwent RARP. (ii) Presence of at least one MRI-visible, biopsy-proven tumor (index tumor). (iii) Tumor diagnosed as organ-confined (cT2) based on clinical evaluation.

Exclusion Criteria: (i) Tumors classified as cT3a or higher. (ii) Tumors with no lesion visible on MRI. (iii) Tumors not consistent with the location of clinically significant cancer on MRI and pathology specimens. (iv) Patients who received treatment prior to imaging examination or surgery.

Based on Prostate Imaging-Reporting and Data System (PI-RADS) version 2.1 [7], cT3a tumors were defined as those exhibiting two or more of the following four features: (i) capsular irregularity or bulging, (ii) invasion or asymmetry of the neurovascular bundles, (iii) “filling in” of the retroprostatic angle, and (iv) a TCL > 10 mm in the axial plane. Seventy-two patients meeting these criteria were included in the analysis. Medical charts were retrospectively reviewed to obtain patient data.

We collected information on age, preoperative PSA concentration, Gleason score, preoperative T classification, preoperative MRI findings, and pathological specimens. MRI-TCL, MRI-TCA, and MRI tumor areas were calculated using Osirix MD (Pixmeo SARL, Bernex, Switzerland), while pathological-TCL and pathological tumor area were calculated using ImageJ® (National Institutes of Health, USA). Axial scans of T2-weighted images were used to measure MRI-TCL, which was defined as the maximum length of the contour of the index lesion in contact with the prostatic capsule.

Prostate specimens were processed using the modified Stanford protocol by step-section analysis [8]. The index lesion in pathological specimens was defined as the lesion with the highest Gleason score or the largest tumor volume. Pathological-TCL was defined similarly to MRI-TCL as the maximum length of the contour of the index lesion in contact with the prostatic capsule. Tumor volumes and pathological TCA were calculated using the following formulas:

(1) $$\begin{array}{c} \text{MRI tumor volume}\left({\text{cm}}^{3}\right)=\text{Area}\left({\text{mm}}^{2}\right)\times \text{slice thickness}\left(3\text{ mm}\right)\times \left(\text{number of slices}+1\right)\times \frac{\pi }{6}\times \frac{1}{1000},\\ \text{Pathological tumor volume}\left({\text{cm}}^{3}\right)=\text{Area}\left({\text{mm}}^{2}\right)\times \text{slice thickness}\left(\text{estimated }3\text{ mm}\right)\times \left(\text{number of slices}+1\right)\times \frac{\pi }{6}\times \frac{1}{1000},\\ \text{Pathological}-\text{TCA}\left({\text{mm}}^{2}\right)=\frac{\text{TCL }\left(\text{mm}\right)}{2}\times \text{slice thickness }\left(\text{estimated }3\text{ mm}\right)\times \frac{\text{number of slices}+1}{2}\times \pi . \end{array}$$

**Figure 2:**
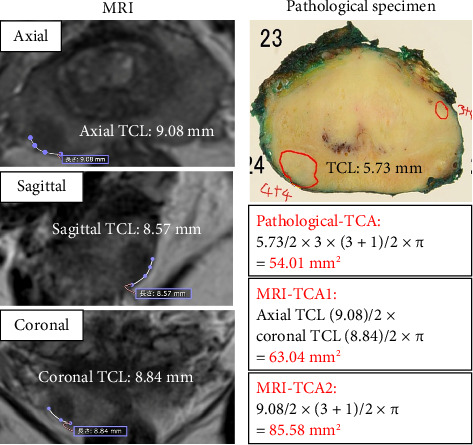
An example of MRI-TCL, MRI-TCA1, MRI-TCA2, pathological-TCL and pathological-TCA.

Although MRI-TCA could be calculated as a portion of the ellipsoid surface area, this method is complex and challenging to apply clinically. Instead, we selected a simplified approach using an elliptical approximation to ensure ease of clinical application. MRI-TCA was calculated using two different methods, both of which allow for practical implementation without requiring specialized image processing or 3D reconstruction software. This approach maintains predictive value while improving accessibility for routine clinical use (Figure 2).

MRI-TCA1: This method approximates the contact area using the TCL in the axial plane and the longer TCL in either the sagittal or coronal plane. It reflects a broader surface area estimate across two planes.

(2) $$\begin{array}{c} \text{MRI}-\text{TCA}1\left({\text{mm}}^{2}\right)=\frac{\text{TCL}\left(\text{mm}\right)}{2}\times \frac{\text{coronal or sagittal TCL}:\text{ longer}}{2}\times \pi . \end{array}$$

MRI-TCA2: This method uses the TCL in the axial plane and the tumor thickness derived from the number of MRI slices. It provides a contact area estimate based on the tumor's extent through multiple slices.

(3) $$\begin{array}{c} \text{MRI}-\text{TCA}2\;\left({\text{mm}}^{2}\right)=\frac{\text{TCL}\left(\text{mm}\right)}{2}\times \text{slice thickness }\left(3\text{ mm}\right)\times \frac{\left(\text{number of slices}+1\right)}{2}\times \pi . \end{array}$$

Patients were stratified according to the optimal thresholds for MRI-TCL, MRI-TCA1, MRI-TCA2, pathological-TCL, and pathological-TCA, and postoperative PSA recurrence-free survival was compared. These optimal thresholds were determined based on Youden's index. Postoperative PSA recurrence was defined as a post-RARP PSA increase to 0.2 ng/mL or higher, measured at least 1 month after surgery.

All statistical analyses were performed using JMP Pro version 16.0.0 (SAS Institute, Cary, NC, USA), with *p* values < 0.05 considered statistically significant. Ordinal and nominal variables were analyzed using Pearson's chi-square test, while continuous variables were analyzed using the Mann–Whitney *U* test. Receiver operating characteristics (ROC) curves were generated to assess the models' ability to predict EPE+, and DeLong's test was used to statistically compare the area under curves (AUC). The comparison of Kaplan–Meier curves was conducted using the log-rank test.

This study was approved by the institutional review board of Kyoto Prefectural University of Medicine (ERB-C-1146-2 and ERB-C-1078-2) and conformed to the provisions of the Declaration of Helsinki. Written informed consent was obtained from all patients. This study followed the Standards for Reporting of Diagnostic Accuracy Studies (STARD) guidelines for diagnostic accuracy reporting.

## 3. Results

Sixteen patients were pathologically EPE+ and 56 were EPE−. The median ages were 69 years for EPE+ patients and 67 years for EPE− patients. The median PSA levels were 7.43 ng/mL for EPE+ patients and 7.17 ng/mL for EPE− patients (Supporting Figure S1). There were no significant differences in Gleason score or index tumor location between the groups. However, the PI-RADS score was significantly higher in EPE+ patients. Additionally, there was no significant difference in the postoperative follow-up duration between the EPE+ and EPE− groups (41.7 months vs. 47.1 months, *p* = 0.8762). Postoperative PSA recurrence was observed in 5 patients (31.25%) in the EPE+ group and 7 patients (12.5%) in the EPE− group. Although the recurrence rate was higher in the EPE+ group, the difference was not statistically significant (*p* = 0.086) (Table 1).

EPE+ tumors had significantly greater areas and volumes than EPE− tumors on both MRI and pathological specimens. On MRI, the median tumor area was 114.85 mm^2^ for EPE+ patients and 63.55 mm^2^ for EPE− patients (*p* = 0.0065), while the median tumor volume was 1.854 cm^3^ and 1.459 cm^3^, respectively (*p* = 0.0213). Similarly, on pathological specimens, the median tumor area was 144.91 mm^2^ for EPE+ and 81.56 mm^2^ for EPE− tumors (*p* = 0.0097), and the median tumor volume was 6.37 cm^3^ and 2.31 cm^3^, respectively (*p* = 0.0037) (Supporting Figure S2).

**Figure 3:**
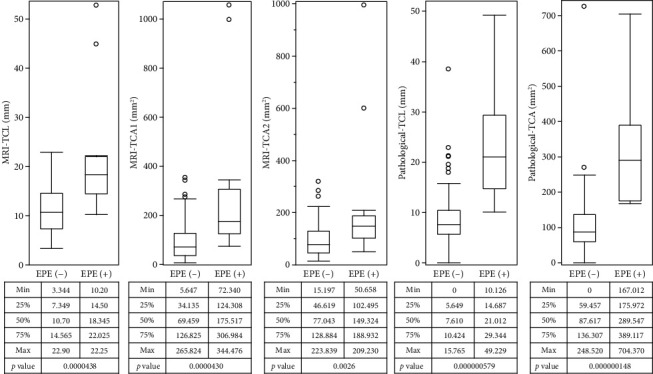
Comparison between EPE− and EPE+ patients in MRI-TCL, MRI-TCA1, MRI-TCA2, pathological TCL and pathological TCA.

MRI-TCL and MRI-TCA (both MRI-TCA1 and MRI-TCA2) were significantly greater in EPE+ patients than in EPE− patients (*p* < 0.001 for all comparisons), with median MRI-TCL values of 14.2 mm vs. 9.5 mm, MRI-TCA1 values of 132.4 mm^2^ vs. 83.7 mm^2^, and MRI-TCA2 values of 107.8 mm^2^ vs. 68.5 mm^2^, respectively. Pathological-TCL and pathological-TCA were also significantly greater in EPE+ patients (*p* < 0.001 for both) (Figure 3).

**Figure 4:**
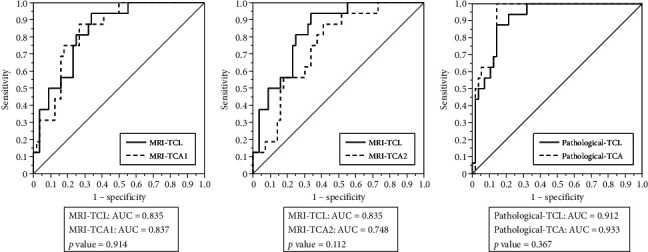
ROC curves were drawn to assess the ability to predict EPE. AUC was drawn to compare between MRI-TCL and MRI-TCA1, MRI-TCL and MRI-TCA2, and pathological-TCL and pathological-TCA.

ROC analysis showed that the AUC for pathological-TCA (0.933, 95% CI: 0.850–0.972) was slightly higher than for pathological-TCL (0.912, 95% CI: 0.819–0.959), though the difference was not statistically significant (*p* = 0.367). This small difference suggests a comparable predictive performance between the two measures. MRI-derived measures had comparable diagnostic accuracy: MRI-TCL (0.835, 95% CI: 0.712–0.912), MRI-TCA1 (0.837, 95% CI: 0.717–0.912), and MRI-TCA2 (0.748, 95% CI: 0.607–0.851). There was no significant difference between MRI-TCL and either MRI-TCA1 (*p* = 0.916) or MRI-TCA2 (*p* = 0.112) (Figure 4), indicating that MRI-TCA offers comparable predictive value without providing additional diagnostic advantage over MRI-TCL.

The optimal thresholds for predicting EPE were 13.0 mm for MRI-TCL (sensitivity: 0.938, specificity: 0.661), 120.174 mm^2^ for MRI-TCA1 (sensitivity: 0.875, specificity: 0.732), and 96.133 mm^2^ for MRI-TCA2 (sensitivity: 0.875, specificity: 0.589).

Spearman's rank correlation coefficients were 0.568 between MRI-TCL and pathological-TCL (*p* < 0.001), 0.561 between MRI-TCA1 and pathological-TCA (*p* < 0.001), and 0.520 between MRI-TCA2 and pathological-TCA (*p* < 0.001) (Supporting Figure S3).

**Figure 5:**
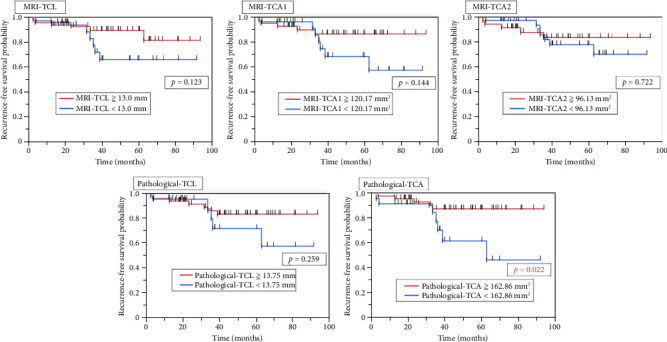
Kaplan–Meier curves for postoperative PSA recurrence-free survival. Comparison of postoperative PSA recurrence-free survival based on the optimal thresholds of MRI-TCL, MRI-TCA1, MRI-TCA2, pathological-TCL, and pathological-TCA.

In the stratified analysis of postoperative PSA recurrence, a significant difference in recurrence rates was observed only for pathological-TCA (*p* = 0.022). No significant difference in postoperative PSA recurrence was found when stratifying by the thresholds for MRI-TCL, MRI-TCA1, MRI-TCA2, or pathological-TCL (Figure 5).

In patients with postoperative PSA recurrence, no significant association was observed between TCL and Gleason score. The Spearman's rank correlation coefficient between MRI-TCL and Gleason score was −0.1979 (*p* = 0.5374), and that between Pathological-TCL and Gleason score was 0.2532 (*p* = 0.4272).

## 4. Discussion

Patients with apparent EPE+ status are at greater risk for positive surgical margins compared to those with EPE− status, and this status is a risk factor for postoperative PSA recurrence [1, 2]. It would be clinically beneficial if we could predict EPE+ more accurately in current MRI-based diagnosed cT2 patients using a reliable quantitative measure.

As a prerequisite, it is crucial that any suspicious clinically significant cancer identified on MRI is confirmed histologically through targeted biopsy before using MRI measurements to predict EPE, as histological verification ensures diagnostic accuracy and prevents misinterpretation of MRI findings.

In our study, pathological-TCA demonstrated a significant ability to predict postoperative PSA recurrence-free survival. Stratification based on pathological-TCA showed a significant difference in postoperative PSA recurrence-free survival, suggesting its potential as a predictor for postoperative outcomes. Moreover, pathological-TCA was useful for predicting EPE.

With prostate MRI, which is known to visualize clinically significant cancer, this study demonstrated that both MRI-TCL and MRI-TCA were significant predictive parameters for pathologic EPE in patients with cT2 prostate cancer. However, contrary to our hypothesis, neither MRI-TCA1 nor MRI-TCA2 demonstrated superiority over MRI-TCL for predicting pathological EPE.

In our study, MRI-TCL was significantly longer in EPE+ patients than in EPE− patients. The optimal MRI-TCL threshold for predicting EPE+ status was 13.0 mm, which was largely consistent with the threshold in PI-RADS version 2.1 for EPE+ [7].

Additionally, EPE+ patients in our study have significantly larger index tumor volumes and higher PI-RADS scores. Similarly, Sugano et al. reported that the index tumor volume on MRI was an independent predictor of EPE+ (*p* = 0.01) [9], while Kim et al. found that the PI-RADS score was a potential predictor of EPE+ (*p* = 0.017) [10]. These findings align with several previous studies, and our results for index tumor volume and PI-RADS are consistent with these prior reports [11–14].

Most importantly, both MRI-TCL and MRI-TCA demonstrated fair diagnostic accuracy in predicting EPE. MRI-TCL and MRI-TCA were significantly correlated with pathological-TCL and pathological-TCA, respectively. Additionally, we observed significant correlations between MRI-TCL and pathological-TCL, MRI-TCA1 and pathological-TCA, and MRI-TCA2 and pathological-TCA. Although MRI-TCA, as calculated in this study, achieved a comparable ability to predict EPE compared to MRI-TCL in both MRI and pathological specimens, it did not surpass MRI-TCL. This may be due to the calculation formula of MRI-TCA used in this study, the limitations of current MRI resolution, and relatively thick slices. Should advancements in MRI technology enable MRI-TCL and MRI-TCA to approach the accuracy of pathological-TCA, these imaging methods could become valuable tools for predicting both EPE and PSA recurrence.

Previous research has explored alternative methods for assessing TCA. Caglic et al. reported that the 3D-converted tumor contact surface, calculated using 3D-conversion software, had better predictive validity than axial TCL (*p* = 0.01) [5]. Similarly, Veerman et al. demonstrated that the 3D-reconstructed contact surface area on mpMRI could predict pathological EPE+ in RP patients (*p* < 0.001) [6]. While these methods offer strong predictive validity, their adaptation to other MRI devices remains challenging due to differences in imaging protocols and software compatibility. In contrast, the MRI-TCA calculation method used in this study is based on a simple formula using the axial plane, slice number, and slice thickness. This approach is clinically simple, highly versatile, and does not require specialized 3D-conversion software or complex image processing.

Ukimura et al. previously reported that TCL, defined as the amount of prostate cancer in contact with the prostatic capsule, correlated better with microscopic EPE than with cancer volume (chi-square 89 vs. 63) in a regression analysis of 189 RP specimens [3]. However, the clinical utility of TCL determined by transrectal ultrasound (TRUS) is limited due to its high operator dependence and poor correlation between TRUS-visualized lesions and pathological cancer lesions. Our study is the first to demonstrate that MRI-TCA, calculated using simple formulas, not only shows a significant correlation with both pathological-TCA and pathological EPE but also highlights the potential of MRI-TCA as a clinically useful predictor for pathological EPE. By combining MRI-TCA with other clinical parameters, such as PSA levels and Gleason scores, MRI-TCA could help refine decision-making for treatment strategies, particularly in guiding surgical planning (whether nerve-sparing or not) and determining the need for adjuvant therapies. However, further validation in larger cohorts is necessary to confirm its clinical utility.

In our study, all patients underwent 3 T MRI without an endorectal coil. However, in clinical practice, 1.5 T MRI is still widely used. Although image quality and spatial resolution are generally inferior to those of 3 T MRI, similar evaluations of MRI-TCA and MRI-TCL may be feasible with optimized imaging protocols and the use of an endorectal coil, albeit with potential limitations in patient comfort and increased artifact. Previous studies comparing 1.5 T MRI with an endorectal coil to 3 T MRI without an endorectal coil have shown that endorectal coil use can improve image quality and tumor delineation, despite the lower field strength [15].

Our study has several limitations, including its retrospective design. While MRI-TCL and MRI-TCA provide quantitative measures for predicting pathological EPE, there remains a risk of underestimation or overestimation. This is particularly true for MRI-TCA, as it is calculated from multiple measures rather than being a direct measurement like MRI-TCL. Variability among interpreters may also contribute to these discrepancies. However, advancements in MRI technology, including improved resolution and thinner slice thickness, may mitigate these issues in the future. Although MRI-TCA did not demonstrate superior accuracy over MRI-TCL, its simplicity, versatility, and ease of use make it a promising tool that could benefit from future MRI technology improvements to enhance predictive accuracy. If future improvements in MRI technology enhance the accuracy of MRI-TCA, it may contribute to more precise determination of surgical indications for RARP and prediction of postoperative outcomes.

## 5. Conclusion

Both MRI-TCL and MRI-TCA are clinically available quantitative parameters for predicting pathological EPE. However, in our study, MRI-TCA did not demonstrate superior predictive ability compared to MRI-TCL. Pathological-TCA was shown to be a significant predictor of both pathological EPE and postoperative recurrence. Advancements in mpMRI technology, such as thinner slice thickness and higher resolution, may improve the accuracy of MRI-TCA, bringing it closer to the precision of pathological-TCA. This could potentially enhance the predictive accuracy for both preoperative EPE and postoperative recurrence-free survival. If an accurate MRI-TCA, as described above, can be measured using a simple and versatile method, it may aid in more precise decision-making regarding the indications for RP.

## Figures and Tables

**Table 1:** The comparison between EPE+ and EPE− patients characteristics.

		EPE (+) *N* = 16	EPE (−) *N* = 56	*p* value
Gleason score	6	0	3	0.2865
	7	11	46	
	8	2	3	
	9	3	4	
	10	0	0	

Location 1	Apex	3	28	0.0072
	Mid	13	21	
	Base	0	7	

Location 2	Left	7	23	0.0781
	Right	9	33	

Location 3	PZ	9	33	0.848
	TZ	7	23	

PI-RADS	3	1	17	0.0013
	4	7	33	
	5	8	6	

Postoperative PSA recurrence	(−)	11 (68.75%)	49 (87.50%)	0.086
	(+)	5 (31.25%)	7 (12.5%)	

Postoperative follow-up duration (months)		41.7 (12–91.8)	47.1 (12.7–107)	0.8762

## Data Availability

The data that support the findings of this study are available on request from the corresponding author. The data are not publicly available due to privacy or ethical restrictions.

## Supporting Information

Supporting Figure S1 shows the comparison between EPE− and EPE+ patients in age and initial PSA using the Mann–Whitney *U* test.

Supporting Figure S2 presents the comparison between EPE− and EPE+ patients in MRI tumor area, MRI tumor volume, pathological tumor area, and pathological tumor volume using the Mann–Whitney *U* test.

Supporting Figure S3 demonstrates the correlations of MRI-TCL with pathological-TCL, MRI-TCA1 with pathological-TCA, and MRI-TCA2 with pathological-TCA, assessed using Spearman's rank correlation coefficient.
