# Supplementary material for: MRI-Determined Tumor Contact Area as a Predictor of Pathological Extraprostatic Extension in Clinical T2 Prostate Cancer
Source: Prostate Cancer. 2025 Oct 26;2025:9165949. doi: 10.1155/proc/9165949 (PMC12580032; doi:10.1155/proc/9165949)
Supplement: Supporting Information — Additional supporting information can be found online in the Supporting Information section. [file 9165949.f1.docx]

**Supplementary Figure S1**

Comparison between EPE- and EPE+ patients in age and initial PSA using Mann-Whitney U test.

**Supplementary Figure S2**

Comparison between EPE- and EPE+ patients in MRI tumor area, MRI tumor volume, pathological tumor area and pathological tumor volume using Mann-Whitney U test.

**Supplementary Figure S3**


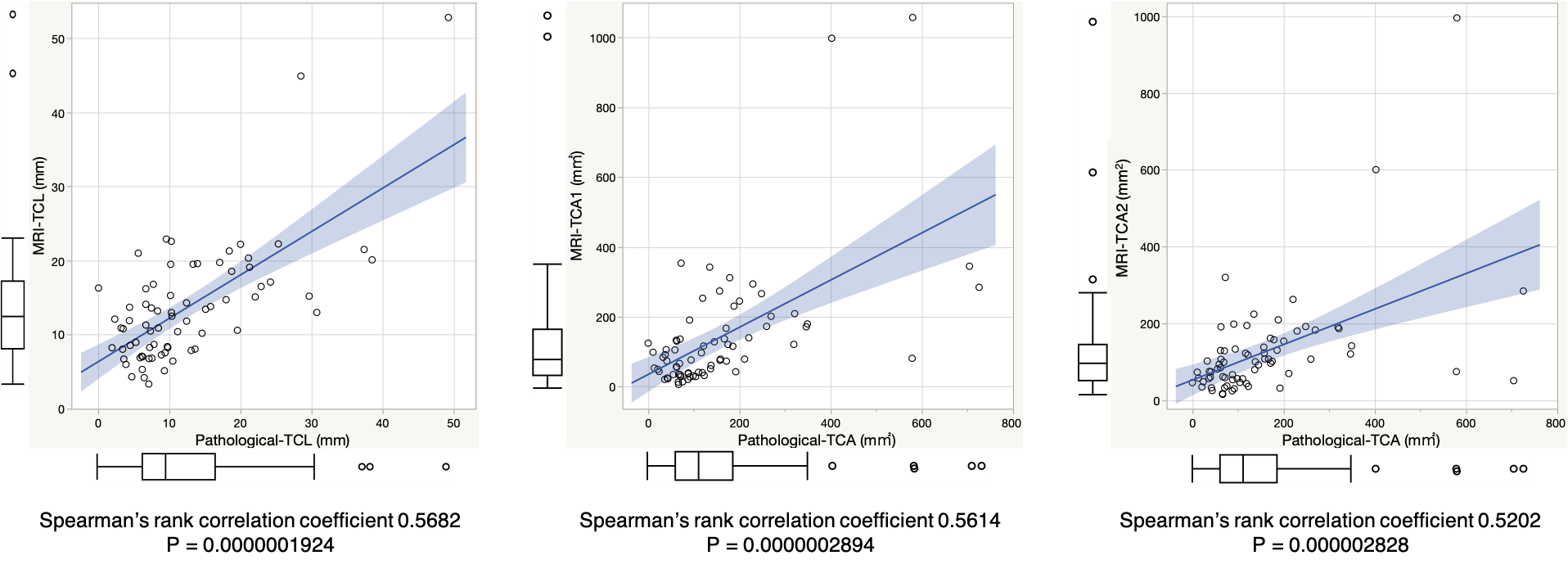


Spearman’s rank correlation coefficient between MRI-TCL and pathological-TCL, MRI-TCA1 and pathological-TCA, and MRI-TCA2 and pathological-TCA.
